# Concomitant Infections With Canine Parvovirus Type 2 and Intracellular Tick-Borne Pathogens in Two Puppy Dogs

**DOI:** 10.3389/fvets.2022.964177

**Published:** 2022-07-19

**Authors:** Lorenza Urbani, Alessandro Tirolo, Andrea Balboni, Roberta Troia, Francesco Dondi, Mara Battilani

**Affiliations:** Department of Veterinary Medical Sciences, Alma Mater Studiorum–University of Bologna, Bologna, Italy

**Keywords:** co-infection, dog, *Ehrlichia canis*, *Hepatozoon canis*, parvovirus enteritis, vector-borne disease

## Abstract

In this report the concomitant infection with canine parvovirus type 2 (CPV-2), *Hepatozoon canis* and *Ehrlichia canis* in two puppy dogs from Southern Italy is described. Dogs were referred to a veterinary university hospital for the acute onset of lethargy and gastrointestinal signs. A complete clinical and clinicopathological evaluation was carried out and the multiple infection was confirmed by microscopic detection of inclusion bodies in peripheral blood smear, rapid immunoenzymatic tests, indirect fluorescent antibody tests, and molecular assays. Sequence analysis revealed that the CPV-2 identified belonged to the 2c variant and had amino acid residues in the predicted VP2 protein typical of “Asian-like” strains widespread in Asia and occasionally reported in Romania, Nigeria and Italy, particularly in the region of Sicily. Numerous monocytes were infected by both *H. canis* gamonts and *E. canis* morulae, suggesting that this co-infection is not accidental and that *E. canis* preferably infects those cells parasitized by *H. canis*. The clinical presentation of these animals was severe but supportive cares associated with early etiological therapy allowed a good prognosis. Movement of puppies from geographic areas where vector-borne pathogens are endemic must be carefully evaluated and core vaccinations and ectoparasite prevention treatments must be rigorously adopted.

## Introduction

Canine parvovirus type 2 (CPV-2) (family *Parvoviridae*, genus *Protoparvovirus*, species Carnivore protoparvovirus 1) is a small, non-enveloped, single-stranded linear DNA virus that infects dogs and cats and requires rapidly dividing cells for replication, in particular hematopoietic and intestinal tissues causing severe immunosuppression and hemorrhagic enteritis (1). Multiple infections (parasitic, viral, or bacterial intestinal pathogens) are frequent in the course of canine parvovirosis and are often facilitated by the state of immunosuppression caused by CPV-2, worsening the overall clinical picture (2, 3). Episodes of concomitant infection with two or more pathogens have been commonly reported in dogs (4–7). Canine hepatozoonosis, ehrlichiosis and anaplasmosis are tick-borne diseases caused by the intracellular pathogens transmitted by the brown dog tick *Rhipicephalus sanguineus* and ticks from the genus *Ixodes*. *Hepatozoon canis* is an apicomplexan protozoon that infects neutrophils and monocytes, whereas *Ehrlichia canis* and *Anaplasma phagocytophilum* are rickettsial organisms that infect mononuclear leukocytes (monocytes and macrophages) and neutrophils, respectively. To date, no data on clinical presentation, pathogenicity and response to therapy in co-infections with CPV-2 and tick-borne pathogens were reported in the literature. In this study, a concomitant natural infection with tick-borne pathogens in two ill dogs tested positive for CPV-2 DNA was described. The CPV-2 was genetically characterized.

## Materials and Methods

### Dogs and Sampling

Two cohabiting puppies were referred to the Veterinary University Hospital (VUH) of the Department of Veterinary Medical Sciences, University of Bologna (Northern Italy), in June 2019, for the acute onset of hemorrhagic diarrhea, vomiting, anorexia and depression. Dogs underwent physical examination; fecal and blood samples were collected for clinicopathological evaluation and detection of infectious diseases. Signalment data, history and vaccination status were collected at the time of hospital admission. Blood sampling was carried out by venipuncture, and samples were collected using a vacuum system (Vacutest, Kima, Arzergrande, PD, Italy). The K_3_EDTA samples were used for a complete blood count (CBC) and molecular analyses. Serum samples underwent a chemistry profile and serum protein electrophoresis. The urine samples were collected by spontaneous voiding for complete urinalysis. All clinicopathological evaluations were carried out within 1 h from specimens' collection, and the samples were stored at −80°C after examination. Fecal, blood and serum samples for infectious diseases investigation were stored at −20°C until use.

### Clinicopathological Investigations

CBC was carried out using an automated hematology analyzer (ADVIA 2120, Siemens Healthcare Diagnostics, Erlangen, Germany). The hematology was completed with the microscopic blood smear examination using May-Grünwald Giemsa staining. Serum chemistry profile, including creatinine, urea, phosphate, total protein, albumin, albumin to globulin ratio (A:G), alanine aminotransferase, aspartate aminotransferase (AST), alkaline phosphatase, gamma (γ)-glutamyltransferase, total bilirubin, cholesterol, total calcium, sodium, potassium, chloride, magnesium and glucose, as reported previously by Troìa et al. (8), was carried out using an automated analyzer (AU480; Beckman Coulter-Olympus, Brea, CA, USA). The coagulation profile included prothrombin time (PT) and activated partial thromboplastin time (aPTT) and was performed using a benchtop analyzer (BFT II, Siemens, Munich, Germany). Urinalysis included urine specific gravity (USG) measured by a hand refractometer (American Optical, Buffalo, New York), dipstick evaluation (Combur10TestUX; Roche, Basel, Switzerland) and microscopic sediment examination. Urinary protein and creatinine were also evaluated (AU480; Beckman Coulter-Olympus, Brea, CA, USA) and the urine protein to creatinine ratio (UPC) was calculated.

### Diagnosis of Infection by Serological, Antigenic and Molecular Tests

A rapid immunoenzymatic test for CPV-2 antigen detection (SNAP PARVO, IDEXX Laboratories, Westbrook, ME, USA) was carried out on fecal samples. Moreover, a rapid immunoenzymatic test for the simultaneous detection of *Dirofilaria immitis* antigen and antibodies against *E. canis* or *ewingii, A. phagocytophilum* or *platys*, and *Borrelia burgdorferi* (SNAP 4DX, respectively, IDEXX Laboratories, Westbrook, ME, USA) was carried out on blood specimens, following the manufacturer's instructions.

*Ehrlichia canis, A. phagocytophilum, Leishmania infantum* and *Toxoplasma gondii* indirect fluorescent antibody tests (IFAT) for IgG (MegaFLUO EHRLICHIA canis, MegaFLUO ANAPLASMA phagocytophilum, MegaFLUO LEISH, MegaFLUO TOXOPLASMA g, MegaCor Diagnostik, Hoerbranz, Austria) were performed on serum samples, following the manufacturer's instructions. Briefly, slides coated with *E. canis, A. phagocytophilum, L. infantum* and *T. gondii* infected cells were probed with sera serially diluted in phosphate-buffered saline (PBS) starting with a concentration of 1:40 until reaching a concentration of 1:2560, incubated for 30 min at 37°C and washed two times with PBS. Internal canine positive and negative sera controls were included on each slide. The slides were probed with 20 μL of fluorescein isothiocyanate (FITC) conjugated anti-dog IgG antibody diluted in PBS at a concentration of 1:64 (Anti-Dog IgG-FITC antibody produced in rabbit; Sigma-Aldrich, Saint Louis, MO, USA) for 30 min at 37°C and were washed two times with PBS and examined under a fluorescent microscope. The highest dilution showing fluorescence was the final antibody titer.

DNA and RNA were extracted from blood samples using the NucleoSpin Tissue Kit (Macherey-Nagel, Düren, Germany) and the QIAamp Viral RNA Mini Kit (Qiagen, Hilden, Germany), respectively, according to the manufacturer's instructions. The extracted DNA and RNA were stored at −20 and −80°C, respectively. Nucleic acids were not extracted from feces because samples were not stored for molecular analysis.

The detection of Carnivore protoparvovirus 1 and all known *Ehrlichia* spp. and *Anaplasma* spp. DNA was carried out with two previously described end-point PCR assayes (9, 10) using the Taq DNA Polymerase Kit (Qiagen, Hilden, Germany), according to the manufacturer's instructions. Positive DNA extracts of CPV-2b (lab ID 934/2017) (11) and of *A. phagocytophilum* (lab ID 862/2014) (12) were, respectively, used as positive controls. A no template control, consisting of ultrapure water underwent analysis simultaneously. The complete VP2 gene of Carnivore protoparvovirus 1 was amplified using the primers reported in Table 1. The thermal cycling consisted of an initial denaturation at 94°C for 5 min followed by 40 cycles of denaturation at 94°C for 30 s, annealing at 50°C for 2 min and elongation at 72°C for 2 min and 20 s, followed by a final elongation step at 72°C for 10 min. A fragment of the groEL gene of *Ehrlichia* spp. and *Anaplasma* spp. was amplified using the primers reported in Table 1. The thermal cycling consisted of an initial denaturation at 94°C for 5 min followed by 45 cycles of denaturation at 94°C for 30 s, annealing at 58°C for 30 s and elongation at 72°C for 45 s, followed by a final elongation step at 72°C for 10 min. PCR products were visualized under UV after electrophoresis migration on a 1% agarose gel stained with Midori Green Advance DNA Stain (Nippon Genetics, Düren, Germany).

**Table 1:** Primers used for molecular detection of viral, bacterial and parasitic DNA and viral RNA.

**Pathogen**	**Primer name**	**Primer sequence**	**DNA/RNA fragment amplified**	**References**
CPV-2	VP2_For	5'-ACCAACTAAAAGAAGTAAACCA-3'	VP2 gene (1,885 bp)	(9)
	VP2_Rev	5'-GTAATAAACATAAAAACATAGTAAGTA-3'		
*Anaplasma* spp.	groEL_For	5'-ACTGATGGTATGCARTTTGAYCG-3'	groEL gene (about 600 bp)	(47)
*Ehrlichia* spp.	groEL_Rev	5'-TCTTTRCGTTCYTTMACYTCAACTTC-3'		
CAdV-1 CAdV-2	CAdV-qPCR_For3	5'-CTGASACTGCWATRMCTATATAYATTTCCA-3'	E3 gene (166 bp)	(14)
	CAdV-qPCR_Rev2	5'-GACATAGARACRCAGGACCCAGA-3'		
CanineCV	CaCV-qPCR_For	5'-CTGAAAGATAAAGGCCTCTCGCT-3'	IR between the ends of the two major ORFs (132 bp)	(16)
	CaCV-qPCR_Rev	5'-AGGGGGGTGAACAGGTAAACG-3'		
*Leptospira* spp.	LipL32_For	5'-AAGCATTACCGCTTGTGGTG-3'	LipL32 gene (242 bp)	(13)
	LipL32_Rev	5'-GAACTCCCATTTCAGCGATT-3'		
*Babesia* spp.	B-lsu_For	5'-ACCTGTCAARTTCCTTCACTAAMTT-3'	B-lsu gene (about 150 bp)	(15)
	B-lsu_Rev2	5'-TCTTAACCCAACTCACGTACCA-3'		
CDV	qCDVFor4	5'-GTCGGTAATCGAGGATTCGAGAG-3'	P gene (114 bp)	(17)
	qCDVRev3	5'-GCCGAAAGAATATCCCCAGTTAG-3'		

*CAdV-1, canine adenovirus type 1; CAdV-2, canine adenovirus type 2; CanineCV, canine circovirus; CDV, canine distemper virus; CPV-2, Canine parvovirus type 2; IR, intergenic region; ORFs, open reading frame*.

The detection of canine adenovirus type 1 (CAdV-1) and type 2 (CAdV-2), canine circovirus (CanineCV), *Leptospira* spp. and *Babesia* spp. DNA was carried out with previously described real-time PCR (qPCR) assays (13–16) using the PowerUp SYBR Green Master Mix Kit (Life Technologies, California, USA) and the StepOnePlus Real-Time PCR System (Thermo Fisher Scientific, Life Technologies, USA), according to the manufacturer's instructions. A fragment of the E3 gene of CAdV-1 and 2, a fragment of the intergenic region between the ends of the two major open reading frames of CanineCV, a fragment of the LipL32 gene of *Leptospira* spp. and a fragment of the B-lsu gene of *Babesia* spp. Were, respectively, amplified using the primers reported in Table 1. The thermal cycling of all reactions consisted of 95°C for 5 min and 45 cycles of 95°C for 15 s and 60°C for 1 min.

The detection of canine distemper virus (CDV) RNA was carried out with previously described one-step real-time reverse transcription PCR (RT-qPCR) assay (17) using the EXPRESS One-step SYBR GreenER Kit (Life Technologies, California, USA) and the StepOnePlus Real-Time PCR System (Thermo Fisher Scientific, Life Technologies, USA), according to the manufacturer's instructions. A fragment of the P gene of CDV was amplified using the primers reported in Table 1. The thermal cycling consisted of a reverse transcription step at 50°C for 5 min, followed by 95°C for 2 min and 40 cycles of 95°C for 15 s and 60°C for 50 s.

All the qPCR reactions were carried out testing in duplicate eight 10-fold dilutions of a standard plasmid (pCR4 plasmid, Invitrogen, USA) containing from 1 × 10^0^ to 1 × 10^7^ copies of the target sequence for microliter used as external standards for the construction of the assay standard curve, the DNA or RNA extracts and a no template control. Melting experiments were carried out after the last extension step by a continuous increment from 55 to 98°C and specific melting temperature (Tm) was about 73°C for CAdV-1 and *Babesia* spp., 80°C for CAdV-2, 93°C for CanineCV, 82°C for *Leptospira* spp. and 78°C for CDV. The limit of detection (LOD) of the assays, assessed by testing serial 10-fold dilutions of the recombinant plasmid, was 1 copy of target amplicon/μL for CAdV-1 and 2, *Leptospira* spp., *Babesia* spp. and CDV, and 5 copies of target amplicon/μL for CanineCV. Samples were considered positive if the amplification fluorescence curve increased exponentially, the Tm was specific and the mean of the target copy number obtained from the replicates was greater than the LOD.

### Sequence Analysis

Amplicons of the expected size were purified using the QIAquick PCR Purification Kit (Qiagen, Hilden, Germany) according to the manufacturer's instructions and directly sequenced by Sanger method (BioFab Research, Italy) using both forward and reverse primers. For the sequencing of CPV-2 VP2 gene, a third internal primer was also used (primer 41: 5′- GCCCTTGTGTAGACGC−3′) (18). The nucleotide sequences obtained were assembled and translated into amino acid sequences using BioEdit sequence alignment editor version 7.2.5. The assembled nucleotide sequences were analyzed using the BLAST web interface (Basic Local Alignment Search Tool)^1^ The assembled VP2 nucleotide sequences were aligned with 79 CPV-2 reference sequences available in the GenBank database (GenBank Overview—NCBI)^2^, using the ClustalW method implemented with BioEdit software, and phylogeny was carried out using the MEGA 11 software version 11.0.11 (19). Phylogenetic tree was constructed using Neighbor-Joining method and the Tamura 3-parameters (T92) model with gamma distribution. The robustness of individual nodes on the phylogenetic tree was estimated using 1,000 bootstrap replicates and bootstrap values >80 were indicated at the corresponding node.

## Results

### Clinical and Clinicopathological Findings

**Figure 1:**
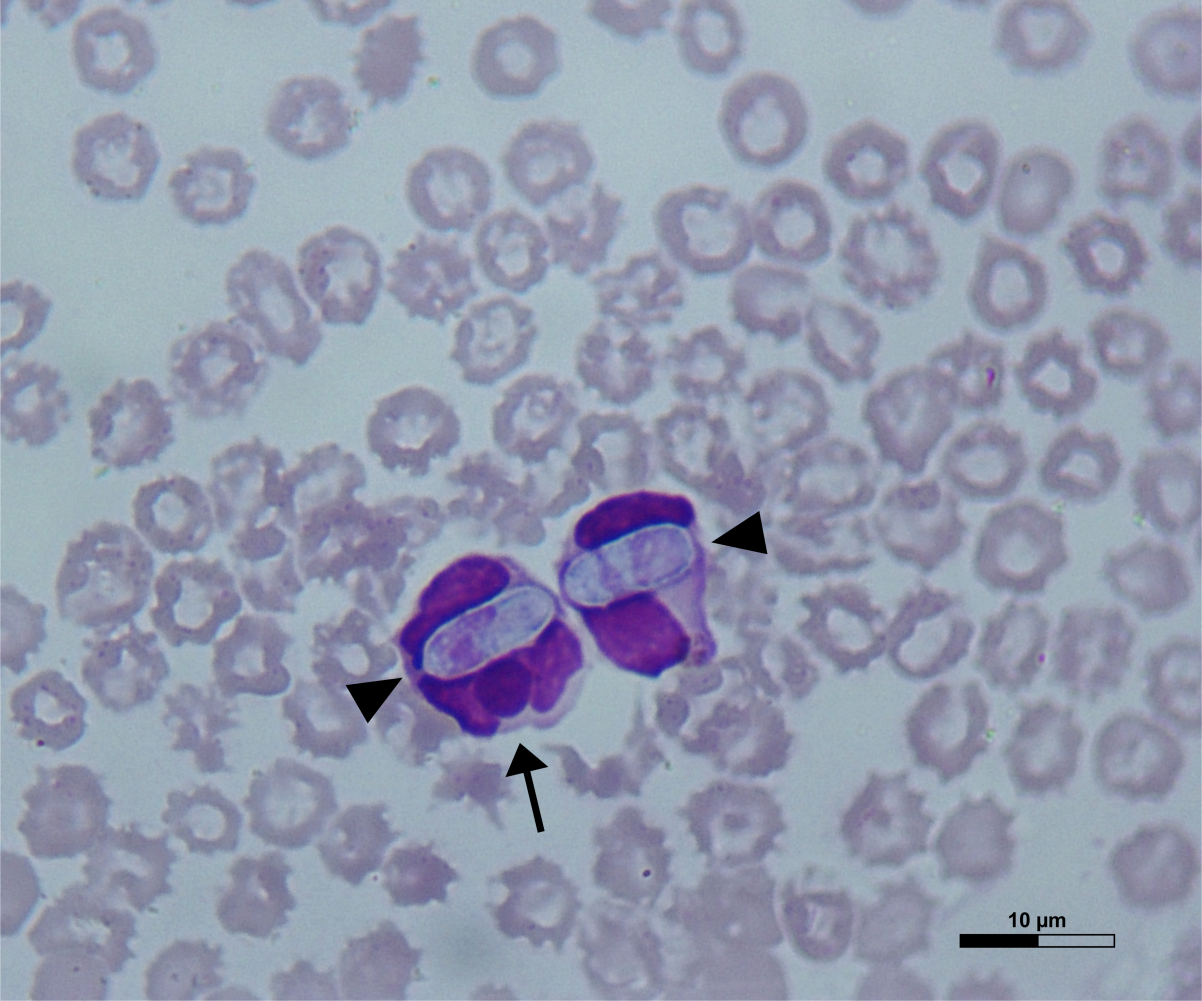
A May-Grünwald Giemsa-stained blood smear from Dog 1 showing *Hepatozoon canis* gamonts (arrowheads) and *Ehrlichia canis* morula (arrow) within a monocyte on the left and a granulocyte on the right.

Dog 1 (lab ID: 636/2019) was a 3-month-old male and Dog 2 (lab ID: 637/2019) was a 5-month-old female, Maremmano-Abruzzese Sheepdogs. Both dogs were unvaccinated and were adopted by a private owner from an animal shelter in the region of Sicily (Southern Italy) and transferred in the region of Emilia Romagna (Northern Italy). Both dogs had gastrointestinal clinical signs such as hemorrhagic diarrhea, vomiting, anorexia and depression. Physical examination revealed a poor nutritional status with a body condition score (BCS) of 4/9 for both dogs, dehydration and the presence of ticks for both dogs. Infectious gastroenteritis (e.g., viral etiology) was suspected by clinicians. CBC showed decreased red blood cell count, hemoglobin concentration and hematocrit value; thrombocytopenia was detected in both dogs. Leukopenia was documented in Dog 1 only (Table 2) at the time of admission, although both dogs became severely leukopenic during hospital stay. Microscopic examination of blood smears showed the presence of *H. canis* gamonts in granulocytes and inclusion bodies morphologically attributable to morulae of *Ehrlichia* spp. or *Anaplasma* spp. in the monocytes of both dogs. Evaluation of 15 fields of the blood smear of each dog indicated that *H. canis* gamonts and morulae were very numerous in Dog 1 and relatively fewer in number in Dog 2, with 46.6 and 4.5% of the leukocytes infected, respectively (Table 2). Some monocytes had both *H. canis* gamonts and morulae (Figure 1).

**Table 2:** Clinicopathological results of Dog 1 and Dog 2.

**Variables**	**Dog 1**	**Dog 2**	**Reference interval**
**Hematology**
RBCs (× 10^6^/μL)	4.9^*^	4.6^*^	5.5–8.5
WBCs (× 10^3^/μL)	2.1^*^	14.3	6–17
Hb (g/dL)	8.8^*^	8.5^*^	12–18
Hct (%)	29.7^*^	27.7^*^	37–55
Platelets (× 10^3^/μL)	45^*^	143^*^	160–500
**Blood smear evaluation**
N. of leukocytes (%) with *Hepatozoon canis* gamonts	24/58 (41.4)	4/112 (3.6)	–
N. of leukocytes (%) with *Ehrlichia canis* morulae	0/58 (0)	0/112 (0)	–
N. of leukocytes (%) with concomitant *H. canis* gamonts and *E. canis* morulae	3/58 (5.2)	1/112 (0.9)	–
**Serum chemistry**
ALT (U/L)	68^*^	28	15–65
AST (U/L)	148^*^	103^*^	15–52
ALP (U/L)	177	151	12–180
GGT (U/L)	2.7	1.7	0–5
Total bilirubin (mg/dL)	0.17	0.14	0.07–0.33
Cholesterol (mg/dL)	167	165	123–345
Glucose (mg/dL)	73	93	65–115
Albumin (g/dL)	1.56^*^	1.20^*^	2.75–3.85
Total protein (g/dL)	5.92	4.51^*^	5.6–7.3
A:G (g:g)	0.36^*^	0.36^*^	0.75–1.35
Creatinine (mg/dL)	0.38^*^	0.37^*^	0.75–1.4
Urea (mg/dL)	35.19	30.78	17–48
Phosphate (mg/dL)	7.17^*^	7.46^*^	2.65–5.40
Potassium (mEq/L)	4.4	5.2^*^	3.8–5.0
Sodium (mEq/L)	143	141^*^	143–151
Chloride (mEq/L)	115.8	111.4	108–118
Magnesium (mg/dL)	1.60^*^	1.51^*^	1.70–2.35
Total Calcium (mg/dL)	9.1^*^	9.4	9.3–11
**Coagulation profile**
PT (s)	7.1	6.3^*^	6.5–8.9
aPTT (s)	13.7	22.2^*^	8.0–16.5
**Urinalysis**
pH	8	6	
USG	1,038	1,054	>1,030
Leukocytes (cells/μL)	Negative	75^*^	Absent
Protein (mg/dL)	Negative	30^*^	Absent
Glucose (mg/dL)	Negative	Negative	Absent
Ketones (mg/dL)	Negative	Negative	Absent
Bilirubin (mg/dL)	Negative	Negative	Absent
Hemoglobin (cells/μL)	Negative	Negative	Absent
UPC	0.64^*^	1.57^*^	0.0–0.5

* *Values outside the reference interval. A:G, albumin to globulin ratio; ALP, alkaline phosphatase; ALT, alanine aminotransferase; aPTT, activated partial thromboplastin time; AST, aspartate aminotransferase; GGT, gamma-glutamyl transferase; Hb, hemoglobin; Hct, hematocrit value; PT, prothrombin time; RBCs, red blood cells; UPC, urine protein:creatinine ratio; USG, urine specific gravity; WBCs, white blood cells*.

Chemistry results showed decreased serum concentration of creatinine and albumin, a decreased A:G, and increased activity of AST, in both dogs. Dog 2 had also decreased serum concentration of total proteins (Table 2). The coagulation profile showed an increase in aPTT in Dog 2 (Table 2). Results of USG and dipstick are shown in Table 2. The UPC was increased in both dogs (Table 2).

### Diagnosis of Infection

Dogs tested positive to CPV-2 antigen and antibodies against *E. canis* or *ewingii* and *A. phagocytophilum* or *platys* by rapid immunoenzymatic tests. IFAT revealed antibody titers ≥1:2560 against *E. canis* and of 1:80 against *A. phagocytophilum* for both dogs. IFAT results for *L. infantum* and *T. gondii* were negative in both sera. Blood samples of both dogs tested positive for Carnivore protoparvovirus 1 and *Ehrlichia* or *Anaplasma* species DNA, while they were negative for CAdV-1, CAdV-2, CanineCV, *Leptospira* spp., *Babesia* spp., and CDV nucleic acids.

### Sequence Analysis

**Figure 2:**
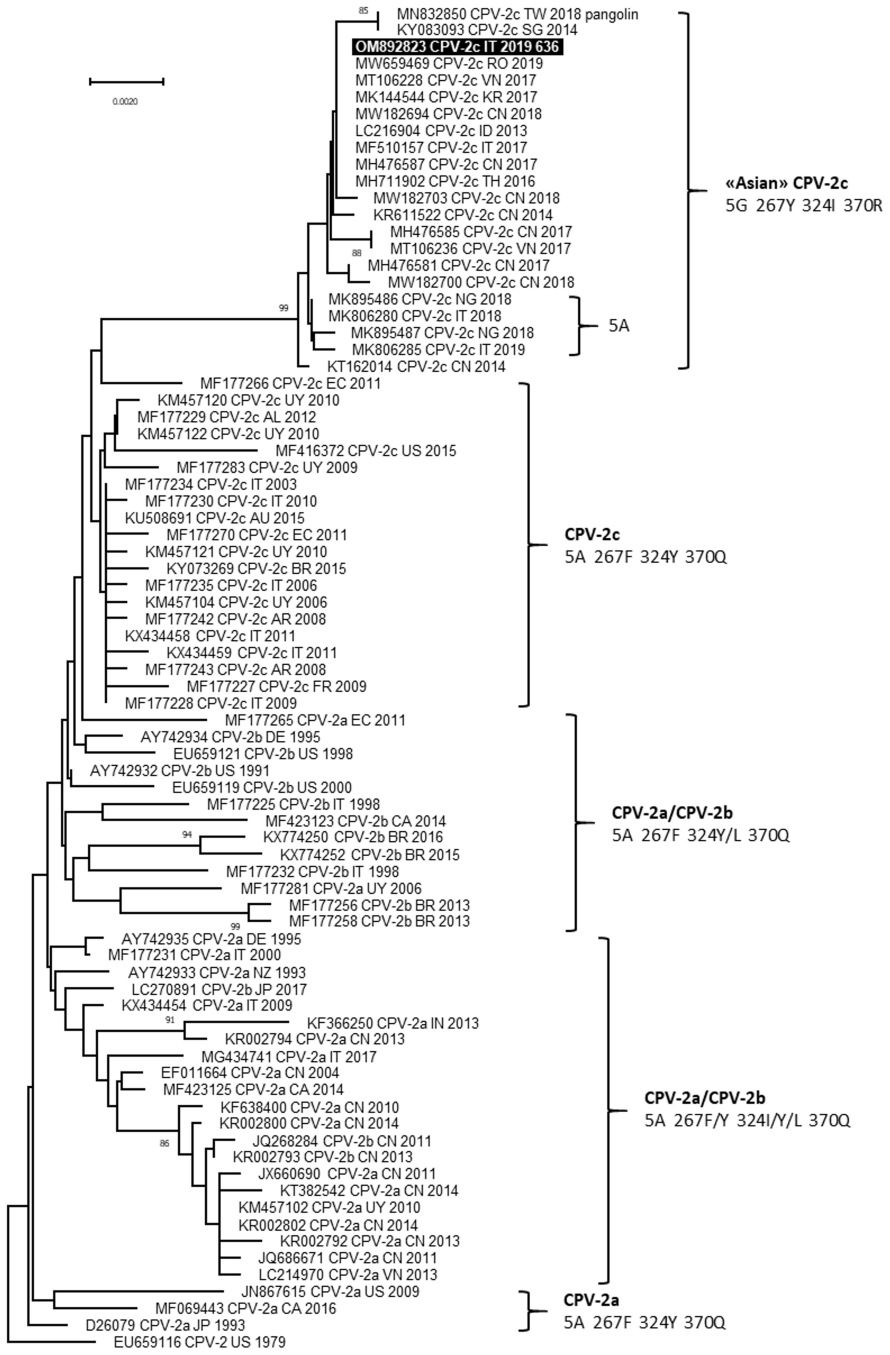
Phylogenetic tree based on the complete VP2 gene nucleotide sequences of canine parvovirus type 2 (CPV-2) obtained in this study and reference strains retrieved from GenBank database. Phylogeny was constructed by MEGA 11 version 11.0.11 using Neighbor-Joining method and the Tamura 3-parameters (T92 model with gamma distribution. Statistical support was provided by bootstrapping with 1,000 replicates. Bootstrap values >80% are indicated on the respective branches. Highlighted in black: Sequences generated in this study (only the viral sequence identified in Dog 1 is reported because the nucleotide sequences obtained from the two dogs were identical). The CPV-2 antigenic variants and the amino acid residues in positions 5, 267, 324 and 370 of deduced VP2 protein are reported for each cluster.

The complete nucleotide sequence of VP2 gene (1,755 nucleotides) was obtained for carnivore protoparvoviruses identified in blood samples of the two dogs and the sequences were identical (GenBank ID: OM892823 and OM892824). Based on the critical amino acid residues of the deduced VP2 protein, the viruses identified were classified as CPV and belonged to the 2c variant, owing to the occurrence of the amino acid glutamate in position 426 (codon GAA) (20). BLAST analysis allowed to identify 60 reference sequences of CPV-2c with full query coverage and complete nucleotide identity and several other CPV-2c reference sequences showing nucleotide identity ≥99.89%, reported from 2013 to 2021 in several Asian regions, Italy, Romania and Nigeria (9, 21–33). All these reference CPV-2c strains and both CPV-2c identified in this study were characterized by distinctive amino acid residues in the deduced VP2 protein: 5-glycine (5G), 267-tirosine (267Y), 324-isoleucine (324I) and 370-arginine (370R). Phylogenetic tree showed a monophyletic cluster, supported by a high bootstrap value, which groups the CPV-2c identified in this study with the other CPV-2c reference strains characterized by the VP2 amino acid residues 5G (or Alanine—A), 267Y, 324I, and 370R (Figure 2).

A partial nucleotide sequence of groEL gene (571 nucleotides) was obtained from blood samples of the two dogs and the sequences were identical (GenBank ID: ON245149 and ON245150). BLAST analysis allowed to identify them as belonging to *E. canis* genome finding full query coverage and complete nucleotide identity with 11 *E. canis* reference strains and nucleotide identity ≤ 81 and ≤ 77% with *Candidatus Neoehrlichia* spp. and *A. phagocytophilum* reference strains, respectively.

### Therapy and Follow Up

Both dogs were hospitalized at the VUH for 10 days. Supportive therapy with intravenous fluids, omeprazole (1 mg/kg IV BID), maropitant (1 mg/kg IV SID) and piperacillin tazobactam (50 mg/kg IV QUID) was initially set. Two transfusion treatments with fresh frozen plasma (FFP) were required during hospitalization in both dogs (Dog 1: 17.4 and 19.9 mL/kg, Dog 2: 11.4 and 19.5 mL/kg). For the treatment of *H. canis* infection, imidocarb-dipropionate (5 mg/kg IM) was administered, and repeated every 3 weeks (3 administrations total) until gamonts were no longer detected on the blood smear and buffy coat smear examination. Based on the suspect of canine ehrlichiosis, therapy with doxycycline was administered (10 mg/kg/die PO for 28 days). After discharge, dogs underwent multiple rechecks showing progressive improvement of clinical and clinicopathological abnormalities. Based on a telephone interview, both dogs were asymptomatic 16 months after discharge.

## Discussion

The two dogs included in this study had clinical signs and had clinicopathologic abnormalities compatible with concomitant CPV-2 and intracellular vector-borne pathogens infections.

Anemia, leukopenia and thrombocytopenia are commonly reported in the acute phase of CPV-2 infection, resulting from the combination of severe inflammation, gastrointestinal bleeding and depletion of hematopoietic cell lines due to the virus effect on the bone marrow (3, 34). Chemistry changes usually include hypoalbuminemia, hypoproteinemia and variable electrolytes abnormalities due to gastrointestinal losses, inflammation and malnutrition (34). Hematologic abnormalities are usually the hallmark of vector-borne diseases in dogs, including anemia and thrombocytopenia; these infections can be more often associated with leucocytosis, neutrophilia and monocytosis (35). Although renal involvement is frequently reported in canine ehrlichiosis, renal function of both dogs was normal and associated with appropriately increased USG. However, both dogs had UPC results above the threshold of 0.5 (Dog 1: 0.64 and Dog 2: 1.57), and particularly in Dog 2 a glomerular involvement could have been suspected. Overall, signalment, history, clinical signs and hematology results of these two dogs led to complete the screening for multiple pathogens.

CPV-2 antigen was detected in fecal samples by rapid immunoenzymatic test and CPV-2 DNA was detected in blood samples of both dogs, suggesting an acute stage of infection. The viruses identified in the two dogs belonged to CPV-2c variant, had identical VP2 gene nucleotide sequences, and showed distinctive amino acid residues in the deduced VP2 protein: 5G, 267Y, 324I, and 370R. Previous studies reported a predominance of the CPV-2c variant in dogs from the region of Sicily (11, 36). To date, viruses with these distinctive amino acid residues in the deduced VP2 protein, recognized as “Asian CPV-2c” because detected for the first time in Asia, are widespread in Asia and occasionally reported in Romania, Nigeria and Italy, particularly in Sicily (9, 21, 23, 26, 28, 30). The close correlation between these CPV-2c is also evidenced by the phylogenetic analysis of the VP2 gene, suggesting the spread of viruses with this distinctive amino acid profile in the worldwide canine population, that continue to acquire mutations such as the amino acid change glycine to alanine in residue 5 (26, 28), as previously assumed (9, 26).

*Hepatozoon canis* gamonts and inclusion bodies morphologically attributable to morulae of *Ehrlichia* spp. or *Anaplasma* spp. were detected in the blood smears of the two dogs. Microscopic examination of the blood smear for the detection of *Ehrlichia* spp. and *Anaplasma* spp. has low specificity; a positive result is rarely seen, but considered strongly specific for the state of active infection in the acute stage (37, 38). Although it is not possible to distinguish between the morulae of *Ehrlichia* spp. and *Anaplasma* spp. because they appear morphologically identical, the type of blood cells infected can be informative, as *E. canis* typically infects monocytes while *A. phagocytophilum* infects neutrophils. In both dogs, morulae were detected in monocytes, primarily indicating an *E. canis* infection. A definitive diagnosis of *Ehrlichia* spp. and *Anaplasma* spp. infection is usually based on antibody detection (rapid immunoenzymatic tests or IFAT), molecular assays on blood samples, or both. In this study, although both dogs tested positive for antibodies against *E. canis* or *ewingii* and *A. phagocytophilum* or *platys* by rapid immunoenzymatic test, the high antibody titer detected by IFAT for *E. canis* (≥1:2560 in both dogs) suggests an active infection, whereas the low antibody titer detected for *A. phagocytophilum* (1:80 in both bogs) could be justified by a cross-reaction with *E. canis* (39). Analysis of the partial groEL gene nucleotide sequences obtained from both dogs by PCR targeting all known *Ehrlichia* spp. and *Anaplasma* spp. DNA matched with *E. canis* genomes, showing complete identity in BLAST analysis. These findings suggest that the two dogs were likely infected by *E. canis*, rather than *A. phagocytophilum*. This finding is not surprising since *E. canis* is the most common tick-borne pathogen identified in Southern Italy (40).

It can be assumed that in the two dogs, clinical findings and laboratory abnormalities were attributable to the synergism of the coexisting CPV-2, *H. canis* and *E. canis* (37). The two dogs probably had acute CPV-2 and *E. canis* infection, while it is not possible to assess the stage of *H. canis* infection and which of the three pathogens infected the two dogs first. Nevertheless, in both dogs, numerous monocytes were infected with *H. canis* gamonts and *E. canis* morulae, suggesting that this co-infection is not accidental and possibly that infection with one pathogen allows or enhances cell invasion by the other. *H. canis* and *E. canis* are both transmitted by *R. sanguineus*, that is considered the most prevalent tick species in Southern Italy (41, 42). The infection of dogs occurs by different routes: *H. canis* is transmitted by oral ingestion of infected ticks containing mature parasite sporozoites, whereas *E. canis* is transferred *via* tick saliva during a blood meal (43, 44). Therefore, infection of dogs by both pathogens simultaneously is not necessarily expected, but several studies supposed that *H. canis* infection might enhance cell invasion by other vector-borne pathogens or could potentiate the pathogenicity of other microorganisms, such as *Leishmania infantum, E. canis*, and *Mycoplasma haemocanis* (6, 40, 45, 46). The presence of *E. canis* morulae in monocytes already infected by *H. canis* suggest a preference of *E. canis* to infect cells parasitized by *H. canis*, as previously assumed by Baneth and colleagues (6). The same Authors reported several possible mechanisms that may facilitate invasion of host cells or increased intracellular survival of these organisms. These include: (i) damage of the host cell outer membrane during infection of one pathogen which may allow invasion of other pathogens; (ii) up-regulation of proteins during infection of one pathogen which may allow the infection of other pathogens, such as up-regulation of a membrane receptor for the pathogen's port of entry; (iii) suppression of some cellular defense mechanisms during infection of one pathogen which may facilitate the entry of other pathogens. Based on the results of the present study, it is possible to assume that the two dogs were initially infected by *H. canis* and later with *E. canis*, and the resulting immune depletion may have favored CPV-2 infection or, alternatively, an initial CPV-2 infection may have further facilitated the onset of *H. canis* and *E. canis* infections.

## Conclusions

Although dogs reported in this study had a severe clinical presentation, an effective diagnosis with a rapid immunoenzymatic test for CPV-2 antigen detection and a microscopic evaluation of the blood smear for the detection of canine hepatozoonosis and a concomitant *Ehrlichia* spp. infection allowed to early and properly treat these animals, resulting in a good prognosis. The possibility of adopting and moving puppies from geographic areas where vector-borne pathogens are endemic must be carefully evaluated, especially when they come from an animal shelter where the risk to contract viral infections is higher. Prophylactic measures, such as core vaccinations and ectoparasite prevention treatments, should be strictly adopted in these animals. Furthermore, it is recommended to test dogs moved from endemic areas for ehrlichiosis and leishmaniosis, as reported in the guidelines on movement and registration of dogs and cats of the Italian Ministry of Health^3^.

## Data Availability Statement

The datasets for this study can be found in the International Nucleotide Sequence Database Collaboration (INSDC; ID: OM892823, OM892824, ON245149, and ON245150).^4^

## Ethics Statement

Ethical review and approval was not required for the animal study because the manuscript has been reviewed by the Animal Welfare Committee of the University of Bologna that confirm that the research described in this manuscript does not fall within Directive 63/2010 of the European Parliament and of the Council on the protection of animals used for scientific purposes (transposed into Italian law by Legislative Decree 26/2014) and thus doesn't require any authorization from the national competent Authorities. The Animal Welfare Committee of the University of Bologna acknowledges that informed consent was given by the owner for each animal, to inform that all examinations and procedures will be performed solely for the patient's health and care, and that the data could have been used for teaching or research. Written informed consent was obtained from the owners for the participation of their animals in this study.

## Author Contributions

LU and AT co-wrote the original manuscript draft and performed laboratory tests. LU, AT, and AB conceived and designed the study and analyzed the data. LU, AT, and RT collected samples and data. AB, RT, FD, and MB reviewed, supervised, and edited the manuscript. All authors read and approved the final manuscript.

## Conflict of Interest

The authors declare that the research was conducted in the absence of any commercial or financial relationships that could be construed as a potential conflict of interest.

## Publisher's Note

All claims expressed in this article are solely those of the authors and do not necessarily represent those of their affiliated organizations, or those of the publisher, the editors and the reviewers. Any product that may be evaluated in this article, or claim that may be made by its manufacturer, is not guaranteed or endorsed by the publisher.

## Footnotes

^1^ https://blast.ncbi.nlm.nih.gov/Blast.cgi

^2^ https://www.ncbi.nlm.nih.gov/genbank/

^3^ https://www.salute.gov.it/portale/caniGatti/dettaglioPubblicazioniCaniGatti.jsp?lingua=italiano&id=2178

^4^ http://www.insdc.org/
